# ANCA Vasculitis and Hemophagocytic Lymphohistiocytosis following a Fecal Microbiota Transplant

**DOI:** 10.1155/2018/9263537

**Published:** 2018-02-18

**Authors:** Adam Amlani, Amy Bromley, Aurore Fifi-Mah

**Affiliations:** ^1^Department of Internal Medicine, University of Calgary, Calgary, AB, Canada; ^2^Department of Pathology, University of Calgary, Calgary, AB, Canada; ^3^Department of Internal Medicine, Division of Rheumatology, University of Calgary, Calgary, AB, Canada

## Abstract

A 69-year-old female with antisynthetase syndrome, a history of multiple recurrent infections, and documented previous negative titres for anti-neutrophil cystoplasmic antibody (ANCA) suddenly developed a de novo MPO-ANCA-associated glomerulonephritis three weeks after a fecal microbiota transplantation (FMT) for recurrent *Clostridium difficile* infections. Six months following her FMT and less than two weeks following treatment for urosepsis, she developed severe cholestasis, a markedly elevated ferritin and hypertriglyceridemia. An initial liver biopsy was suggestive of drug-induced liver injury and thus she was treated with supportive care. After she failed to improve, a second liver biopsy supported the diagnosis of hemophagocytic lymphohistiocytosis (HLH). This case highlights difficulties surrounding the early diagnosis of HLH and also questions the role of FMT and/or recurrent infections as a trigger for ANCA-associated vasculitis.

## 1. Case Report

Triggers of autoimmune dysfunction leading to systemic autoimmune rheumatic diseases are not well understood, classically described as multifactorial with a genetic predisposition [1]. We present a 69-year-old female with antisynthetase syndrome (ASTS) presenting with myalgia, arthritis, interstitial lung disease, mechanics hands, and positive Jo-1 antibody requiring multiple courses of prolonged immunosuppression with corticosteroids, azathioprine, mycofenolate mofetil, and rituximab. This was complicated by *Escherichia coli* urosepsis, sinusitis, shingles, and refractory and recurrent *Clostridium difficile* infections (CDIs). She received a fecal microbiota transplant (FMT) via colonoscopy leading to eradication of CDI.

**Figure 1:**
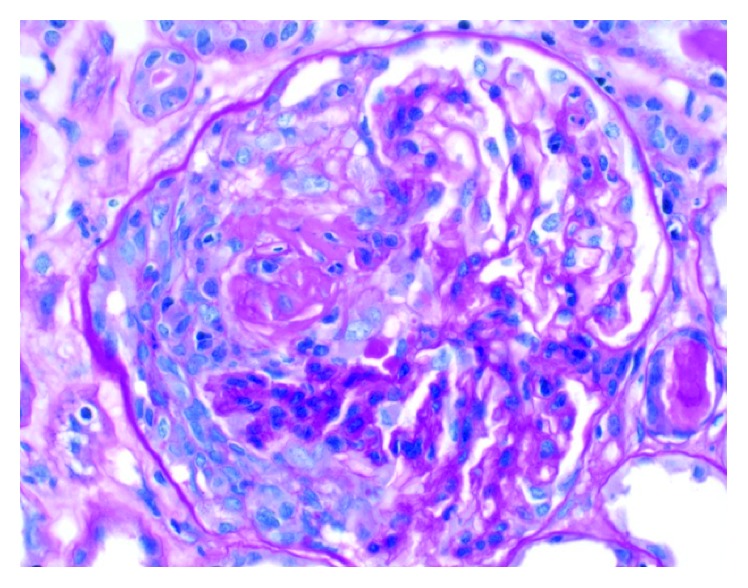
Glomerulus from July 2016 kidney biopsy showing segmental fibrinoid necrosis and a cellular crescent.

Three weeks following the FMT, she developed a severe nephritic syndrome with positive MPO-anti-neutrophil cytoplasmic antibody (ANCA). ANCA had been negative in the past. A renal biopsy revealed a pauci-immune necrotizing and crescentic glomerulonephritis (Figure 1) suggestive of MPO-ANCA-associated nephritis. She was treated with apheresis, cyclophosphamide, and methylprednisolone intravenously and required multiple runs of hemodialysis.

Six months after the development of her ANCA-associated vasculitis (AAV), she was readmitted for 2 weeks for *Escherichia coli* urosepsis. 12 days following her discharge, she re-presented with an acute liver injury. Her stool culture was negative for *C. difficile*. Hemophagocytic lymphohistiocytosis (HLH) was suspected as her ferritin was more than 8000 pmol/L with hypertriglyceridemia at 6.34 mmol/L. A liver biopsy showed hepatocellular dropout, microabscesses, and mild chronic portal inflammation most consistent with a drug-induced liver injury thought to be secondary to a recent course of amoxicillin/clavulanate. She was therefore treated with supportive care.

The patient's bilirubin continued to climb over the next month, peaking at 1034 umol/L. Therefore, a second liver biopsy was performed. Shortly after the procedure, she experienced hemorrhagic shock, cardiac arrest with pulseless electrical activity, and death soon after.

**Figure 2:**
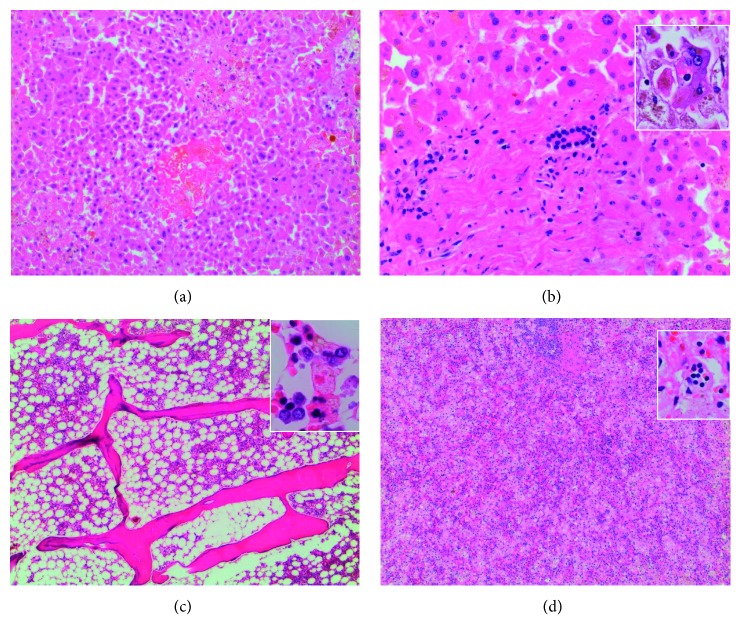
Photomicrographs from autopsy. (a) Liver (40x) with focal necrosis and microabscesses. (b) Liver (100x) with normal bile ducts without inflammation. Inset (400x): phagocytosis of blood cells by Kupffer cells. (c) Bone marrow (40x) displays appropriate cellularity for age with a slight reactive shift. Inset (400x): hemophagocytosis in the bone marrow. (d) Spleen (40x) with sinusoidal expansion by macrophages with abundant hemophagocytosis (inset: 400x).

At autopsy, there was a large hemoperitoneum surrounding her liver secondary to the second biopsy, which was identified as the immediate cause of death. The second liver biopsy, reported postmortem, revealed prominent sinusoidal histiocytosis, patchy hepatocyte necrosis, hemophagocytosis, and hemosiderosis. These findings were not present on the initial liver biopsy described above. The same findings were present on the liver at autopsy, along with hemophagocytosis in the bone marrow and spleen (Figure 2). Therefore, the diagnosis of HLH was confirmed.

## 2. Discussion

ASTS is characterized by the presence of an antiaminoacyl-tRNA synthetase antibody, as well as at least one of interstitial lung disease (ILD) and/or inflammatory myopathy [2]. ASTS has been described within the realm of an overlap syndrome in conjunction with various autoimmune diseases [3]; however, we are unaware of any description of an overlap syndrome of ASTS and an AAV.

Various infections have been labeled as triggers for vasculitis, including urinary and gastrointestinal sources [4]. Unknown antigens have been hypothesized to facilitate the expression of PR3 in neutrophils, enabling the production of anti-PR3 antibodies leading to vessel thrombosis and granuloma formulation [5]. Cases of patients with CDIs and vasculitic processes have been reported; however, it remains relatively unclear as to whether CDIs serve as a clear trigger, risk factor, or consequence of vasculitis [6–9]. The majority of reported cases within this realm describe CDIs in previously diagnosed Henoch-Schönlein purpura (HSP) or IgA-associated vasculitis [7–9]. One case report describes a CDI which may have triggered a limited form of granulomatosis with polyangiitis to evolve systemically within a time course of two weeks [5]. We are unaware of any formerly reported cases in which a CDI actually precedes the development of a de novo AAV, although we cannot exclude the possibility that her recurrent CDIs may have primed her to develop the AAV via mechanisms similar to the above described.

To date, we have not found any evidence supporting FMTs as a trigger for the development of vasculitis, or for the shift from an existing rheumatic autoimmune disease to AAV. It is possible that her AAV was an independent de novo development in a patient who was at a baseline higher risk secondary to her autoimmunity. However, the timeline of the FMT in this case in conjunction with the development of an AAV suggests a possible underlying association. The timeline in this case of three weeks was similar to a previous case in which symptoms of a CDI preceded the development of a HSP by approximately one month [9]. We hypothesize that the biological mechanism would be similar to that of an infection triggering an AAV [5], with the foreign microbiota from the FMT serving as an unknown antigen. If true, to our knowledge, this may be the first reported case of such an association in the literature.

During her last hospitalization, she did have biochemical markers which were suspicious for HLH (fever, hypertriglyceridemia, and markedly elevated ferritin) but otherwise looked relatively well from a clinical perspective. She may not have had histological evidence on the initial biopsy because she was too early in the HLH disease process. This is a known challenge in the timely diagnosis of HLH in adults, although histological evidence of hemophagocytosis is not required for the diagnosis [10].

We have presented a unique case of an overlap between antisynthetase syndrome and MPO-ANCA glomerulonephritis following a stool transplant. This patient ultimately developed HLH that may have been triggered by a recent infection or the development of MPO-ANCA vasculitis. This case highlights the possible role of infection or changes in microbiota as a trigger for ANCA vasculitis and emphasizes the difficulties surrounding the early diagnosis of HLH. Research in the epigenetic influence of environmental triggers in the development of vasculitis is required.
